# Perceptions of basic psychological needs satisfaction, perceived enjoyment, and perceived effort in physical education among middle school students in Saudi Arabia

**DOI:** 10.3389/fpsyg.2026.1974892

**Published:** 2026-10-20

**Authors:** Ali N. Alshuraymi, Peter A. Hastie, Omar Albaloul, Fabian Correia, Kevin Andrew Richards

**Affiliations:** 1 Department of Curriculum and Instruction, College of Education, King Saud University, Riyadh, Saudi Arabia; 2 School of Kinesiology, Auburn University, Auburn, AL, United States; 3 College of Education, Kuwait University, Kuwait City, Kuwait; 4 Department of Teacher Education, College of Education and Human Services, Valdosta State University, Valdosta, GA, United States; 5 College of Applied Health Sciences, Health and Kinesiology, University of Illinois Urban-Champaign, Champaign, IL, United States

**Keywords:** autonomy, competence, motivational climate, relatedness, self-determination theory

## Abstract

**Purpose:**

To examine middle school students' perceptions of basic psychological needs satisfaction, enjoyment, and effort in physical education (PE) in Saudi Arabia.

**Methods:**

A quantitative, cross-sectional, descriptive-comparative design was employed.

Participants were 188 male students aged 11–15 years from four middle schools in Riyadh. Students completed validated measures of autonomy, competence, relatedness, enjoyment, and effort in PE.

**Results:**

Students reported high levels of relatedness, competence, enjoyment, and effort, while perceptions of autonomy were comparatively lower. No significant differences were found by nationality or grade level. However, students who participated in two PE lessons per week reported significantly greater psychological needs satisfaction and enjoyment than those receiving one lesson per week. Basic psychological needs satisfaction was positively associated with both enjoyment and effort.

**Discussion/Conclusion:**

The findings suggest that PE in Saudi middle schools provides an environment that generally supports students' relatedness and competence and is associated with high levels of enjoyment and effort. Autonomy, however, appears to be less well supported. Greater use of autonomy-supportive teaching practices may enhance students' experiences in PE, strengthen alignment with national curriculum goals, and contribute to the development of positive attitudes toward lifelong physical activity.

## 1 Introduction

Physical education (PE) may have an important educational contribution to students' personal development. It provides opportunities for enjoyment, learning new motor skills, and co-operating with others (Hassandra et al., 2003). Research has identified a possible long-term link between current and future physical activity behaviors, such that physically active adolescents are more likely to remain active in adulthood (Conroy et al., 2005). However, 65% of young individuals aged between 11 and 15 years in Europe are physically inactive (Cavill et al., 2006); 60% of U.S. children aged between 9 and 13 years do not participate in any organized PA during non-school hours, and 22.6% of U.S. children do not engage in any free-time physical activity (Rosamond et al., 2008). Although the determinants of physical activity are multifaceted and operate at different levels of influence (Sallis et al., 1998), PE is uniquely positioned to address children's and adolescents' physical inactivity, as the setting affords all children opportunities for physical activity. The same trend was observed in Saudi Arabia, as Al-Hazzaa et al. (2014) reported that self-report and pedometer measures indicated that the majority of Saudi Arabian schoolchildren were inactive.

In recent years, PE in Saudi Arabia has undergone remarkable progress, propelled by nationwide educational reforms aligned with Saudi Vision 2030. These reforms focus on improving educational quality, promoting healthier lifestyles, boosting sports participation, and encouraging lifelong physical activity among youth. The standardized PE curriculum in Saudi Arabia is sport-focused and carefully structured according to grade level, enabling students to progressively develop their skills and understanding. For example, seventh graders learn soccer techniques, including throw-ins, toe stops, dribbling, and stationary kicks (Ministry of Education, 2023a). A typical PE lesson starts with a warm-up, transitions to teacher-led, skill-building activities, and concludes with gameplay involving two balanced, mixed-ability teams to promote inclusivity. The Saudi Arabian middle school curriculum features a range of team sports, such as basketball, handball, volleyball, badminton, and soccer, presented in concise instructional units of four lessons each (Ministry of Education, 2023a,b). The Ministry of Education (MoE) is also committed to developing the expertise of PE teachers and supervisors. Regular training programs are held and viewed as essential for career progression, including promotions. These initiatives aim to cultivate a strong sporting culture, motivate physical activity, and discover and support athletic talent within the community (Ministry of Education, 2023c).

Self-Determination Theory (SDT; Ryan and Deci, 2017) posits that need-supportive environments predict desired motivational outcomes by satisfying people's basic psychological needs, which, in turn, enhance the quality of motivation. According to SDT (Ryan and Deci, 2017), all people have three essential, inherent psychological needs. Research has shown that satisfaction of the needs for autonomy, competence, and relatedness enhances motivation. In educational settings, supporting students' autonomy entails nurturing their intrinsic motivational resources by respecting their attitudes and suggestions (Vasconcellos et al., 2019). This includes providing them with meaningful choices and encouraging initiative-taking (Haerens et al., 2015). Research indicates that students' quality of motivation, autonomous motivation, controlled motivation, and amotivation strongly predict their engagement in PE, participation in physical activity, and persistence (Ntoumanis and Standage, 2009). For example, Sanchez-Oliva et al. (2014) found that higher levels of selfdetermined motivation lead to greater enjoyment, increased perceived importance of PE, and a stronger intention to remain physically active outside of school. When students' psychological needs are satisfied in PE, they experience enhanced psychological, social, and behavioral wellness.

Conversely, psychological need frustration can result in distress, social withdrawal, and diminished behavioral outcomes (Ryan and Deci, 2017). Activities that are overly challenging or paired with frequent negative feedback can undermine students' sense of mastery and hinder the development of competence (White et al., 2021). The lack of student motivation is a considerable challenge for many PE teachers globally, particularly among those who use traditional teaching methods. However, different pedagogical approaches used by PE teachers may have varying effects on student motivation (Spittle and Byrne, 2009). Research has shown that students' motivation plays a significant role in shaping their learning outcomes (Chen, 2001) and in fostering positive behavior in PE and sports (Mitchell, 1996).

According to SDT, the motivation continuum ranges from amotivation to intrinsic motivation. Amotivation refers to a lack of intention and motivation to engage in any activity (Deci and Ryan, 2008). Extrinsic motivation varies in autonomy, from external regulation (driven by external rewards or punishments) and introjected regulation (motivated by guilt or self-worth) to more autonomous forms such as identified regulation (when the activity aligns with personal values) and integrated regulation (when it becomes part of one's self-concept). These motivations are often grouped into autonomous (intrinsic, identified, integrated) and controlled (external, introjected) categories (Cheon et al., 2012). Intrinsic motivation represents the highest level of autonomous motivation (Vasconcellos et al., 2019) and has been a central focus in educational motivation research (Ryan et al., 1985). As defined by Deci and Ryan (1985), intrinsic motivation refers to behaviors undertaken for their inherent enjoyment and satisfaction, driven by the act itself rather than external rewards. Students with intrinsic motivation are more inclined to have favorable PE experiences (Vasconcellos et al., 2019). Studies in sports (Pelletier et al., 1995) and PE (Ntoumanis, 2001) indicate that intrinsic motivation correlates positively with reduced boredom, increased self-effort, and heightened intention to engage in future physical activity among students. Vallerand (2001) argues that providing a learning environment that fulfills all three psychosocial needs is a crucial precursor to increasing student motivation. Within SDT, children's proactive pursuit of basic psychological needs occurs in social contexts that can either support or thwart it (Curran and Standage, 2017). Students who perceived that their PE teacher allocates resources to them and tries to develop strategies to support their autonomy, competence, and relatedness expressed more fulfillment of these psychological needs and were more likely to report that they are involved in PE classes because of self-determined reasons (such as enjoyment, personal importance, and challenge-seeking (Leo et al., 2022).

The PE teacher plays a crucial role in students' motivation. Their teaching behavior can lead to different motivational directions. Autonomy-supporting teachers display patience, allowing students the time they need for self-paced learning, and acknowledge and accept expressions of negative affect (Reeve, 2009). The teacher facilitates autonomy by helping students with their decision-making regarding the choice of practices, which must be inclusive of all members in the small-group structure (Wallhead and Ntoumanis, 2004). It has been shown elsewhere that meaningful choice is an important tenet in the development of pupils' autonomous motivation for PE (Ntoumanis and Standage, 2009). However, it is not only the teacher who can influence children's satisfaction of psychological needs in PE. Interactions with parents, peers, and other macro-level factors (e.g., social norms, school values, and school organization) also influence this (Curran and Standage, 2017). Ensuring that children perceive they have adequate opportunities to develop competencies, self-direct their behaviors, and be connected to others is therefore of paramount importance (Curran and Standage, 2017). It is also imperative to understand that not all children enjoy the same things.

### 1.1 Perceived enjoyment and effort in PE in Saudi Arabia

Alshuraymi and Hastie (2025) compared traditional PE with Sport Education among Saudi middle school students, focusing on perceived enjoyment and effort. Their study found that Sport Education led to significantly higher perceived effort and improvements in both enjoyment and effort, while traditional methods improved only enjoyment. Key features of Sport Education, like persistent teams and formal competition, helped motivate students and sustain their engagement. The model also proved more effective at maintaining intrinsic motivation and achieving Saudi national PE objectives, likely due to its alignment with self-determination theory (SDT; Wallhead and Ntoumanis, 2004; Alshuraymi and Hastie, 2025). Although SDT has been widely applied in PE research, most empirical evidence has been generated in North America, Europe, and other Western educational contexts. Consequently, relatively little is known about students' perceptions of basic psychological needs satisfaction, perceived enjoyment, and perceived effort in PE within Middle Eastern countries. In Saudi Arabia, research examining students' motivational experiences in relation to the national standardized PE curriculum remains limited. Leading to the purpose of this study was to examine middle school students' perceptions of basic psychological needs satisfaction, perceived enjoyment, and perceived effort in PE in Saudi Arabia. Specifically, the study described students' perceptions of autonomy, competence, and relatedness, as well as their perceived enjoyment and perceived effort during PE.

## 2 Materials and methods

### 2.1 Research design

The present study utilized a quantitative, cross-sectional, descriptive-comparative research design without experimental manipulation. Specifically, the study described middle school students' perceptions of basic psychological needs satisfaction, perceived enjoyment, and perceived effort in PE and examined differences in these variables based on nationality, grade level, and the number of PE lessons offered each week.

### 2.2 Participants and settings

The study sample consisted of 188 male middle school students aged 11 to 15 years (*M* = 13.35, *SD* = 0.70) who were recruited from four all-boys middle schools in Riyadh, Saudi Arabia. A convenience sampling approach was employed, whereby schools were recruited through PE teachers who volunteered to participate. Classes were then selected according to teacher availability and the schools' schedules during the data collection period.

Of the participants, 80.3% were Saudi nationals and 19.1% were non-Saudi. The sample included students from Grade 7 (41.5%) and Grade 8 (58.5%). The participating schools represented three school types: two public (general) schools, one public Quranic school, and one private school.

All participating schools implemented the Saudi Ministry of Education's national multi-activity PE curriculum (Ministry of Education, 2023a,b). The frequency of PE instruction differed across school types, with students in the public (general) and private schools receiving two 45-min PE lessons each week, whereas those in the public Quranic school received one 45-min lesson per week. Instruction was delivered by qualified male PE teachers following the teaching practices commonly used in Saudi schools, typically consisting of a teacher-directed warm-up, skill instruction and practice, and game-based activities.

### 2.3 Instruments

#### 2.3.1 The basic psychological needs in physical education scale

Basic psychological needs satisfaction in physical education (BPN-PE) was measured using the physical education adaptation of the Basic Psychological Needs in Exercise Scale developed by Vlachopoulos and Michailidou (2006) and later validated for PE settings by Vlachopoulos et al. (2011). The instrument has demonstrated acceptable psychometric properties among adolescents (Vlachopoulos et al., 2011) and has also shown satisfactory reliability when administered to Saudi Arabian middle school students (Alshuraymi and Hastie, 2025).

The scale includes 12 items introduced by the statement, “In general in PE….” Participants rated each item on a 7-point Likert scale ranging from 1 (completely disagree) to 7 (completely agree), with a midpoint of 4 (moderately agree). The instrument assesses three dimensions of basic psychological needs satisfaction—autonomy, competence, and relatedness—with four items representing each dimension. Example items include “I feel that the way PE is taught is the way I would like it to,” “I feel I do very well even in the tasks considered difficult by most students,” and “I feel like a valued member of a group of close friends.”

Average scores were calculated for each subscale to represent students' perceived autonomy, competence, and relatedness in PE. In addition, an overall BPN-PE score was obtained by averaging responses across all 12 items, with higher scores reflecting greater satisfaction of students' basic psychological needs during PE.

#### 2.3.2 Perceived enjoyment and effort in PE

Students' perceived enjoyment and perceived effort PE were assessed using the

Enjoyment/Interest and Effort/Importance subscales of the Intrinsic Motivation Inventory (IMI; Ryan, 1982), as adapted for sport settings by McAuley et al. (1989). The IMI has demonstrated satisfactory reliability among Arabic-speaking middle school students (Albaloul et al., 2024; Alshuraymi and Hastie, 2025).

Although the original IMI consists of 18 items measuring four dimensions of intrinsic motivation (Enjoyment/Interest, Effort/Importance, Perceived Competence, and Pressure/Tension), only the Enjoyment/Interest and Effort/Importance subscales were used in the present study. These two subscales comprise eight items, with four items assessing perceived enjoyment (e.g., “Playing PE activities is fun”) and four items assessing perceived effort (e.g., “I try very hard while playing PE activities”). Responses were recorded on a 7-point Likert scale ranging from 1 (strongly disagree) to 7 (strongly agree). Mean scores were computed separately for each subscale, with higher scores indicating greater perceived enjoyment and effort in PE.

### 2.4 Translation of the instruments

Given that the study participants were native Arabic speakers likely to struggle with the English versions of the instruments, the first author managed the translation from English to Arabic. Following the guidelines set by Sousa and Rojjanasrirat (2011) for cross-cultural instrument validation, the first author translated, adapted, and validated the instruments to create a culturally equivalent version for Saudi middle school students. The process included the following steps: (a) initial translation by the first author and an Arabic-speaking Ph.D. student; (b) comparison of the translations with the original instruments, resolving any ambiguities; (c) blind back-translation of the English versions into Arabic by two certified English teachers fluent in Arabic; (d) review of the back-translated instruments against the originals to identify discrepancies; and (e) issuance of an official certificate from a certified translation center confirming the translation's accuracy.

### 2.5 Procedure

Data collection took place during the first semester of the 2024–2025 academic year in Saudi Arabia. The questionnaires were administered in person by the first author at each participating school using a standardized administration procedure to ensure consistency across all sites. Before completing the survey, students were informed that the study aimed to examine their perceptions of their current PE experiences. They were also assured that participation was anonymous and that no personally identifiable information would be collected.

Ethical approval for the study was obtained from the Research Ethics Committee at the first author's university and the General Directorate for Education in the Riyadh Region. Following institutional approval, permission to conduct the study was secured from the participating schools. Informed consent was obtained from students and their legal guardians for data collection.

### 2.6 Statistical analysis

Data were analyzed using IBM SPSS (31) Statistics. Descriptive statistics, including means and standard deviations, were calculated to examine students' perceptions of autonomy, competence, relatedness, overall basic psychological needs satisfaction in physical education (BPN-PE), enjoyment, and perceived effort. Internal consistency reliability was examined using Cronbach's alpha for each scale and subscale.

To examine group differences, Welch's independent-samples *t* tests were conducted for nationality, grade level, and weekly PE lesson frequency. Welch's tests were used because several comparisons involved unequal group sizes and because Welch's test does not assume equal population variances. The comparison analyses used the relevant comparison subsamples to reduce school-context confounding. Specifically, nationality comparisons were conducted using students from schools that included both Saudi and non-Saudi students, and grade-level comparisons were conducted using students from schools that included both 7th- and 8th-grade students. Weekly PE lesson comparisons were interpreted cautiously because weekly PE lesson frequency was linked to school context.

For each grouping variable, three outcomes were examined: overall BPN-PE, enjoyment, and perceived effort. Therefore, statistical significance was interpreted using a Bonferroni-adjusted alpha level of 0.017 (0.05/3) within each comparison set. Cohen's *d* was used to estimate effect size and was interpreted using absolute values, with approximately 0.20 considered small, 0.50 medium, and 0.80 large. Pearson product-moment correlations were conducted to examine the relationships among overall BPN-PE, enjoyment, and perceived effort.

The overall BPN-PE score was included because Self-Determination Theory (SDT) conceptualizes autonomy, competence, and relatedness as three distinct but complementary basic psychological needs that collectively contribute to individuals' overall psychological need satisfaction (Ryan and Deci, 2017). Thus, while each need was examined separately to preserve its unique contribution, the overall BPN-PE score was used to provide an aggregate indication of students' general level of basic psychological need satisfaction in PE. This approach is consistent with the SDT perspective that satisfaction of these three needs collectively supports high-quality motivation and positive motivational outcomes.

## 3 Results

**Q1: What are middle school students' perceptions of basic psychological needs satisfaction, enjoyment, and effort in PE in Saudi Arabia?**

Descriptive statistics were calculated for the BPN-PE subscales, the overall BPN-PE score, and the IMI subscales. Internal consistency reliability was also examined using Cronbach's alpha. As shown in Table 1, students reported generally positive perceptions across all variables. Among the BPN-PE subscales, relatedness had the highest mean score (*M* = 5.47, *SD* = 1.38), followed by competence (*M* = 5.32, *SD* = 1.28) and autonomy (*M* = 4.86, *SD* = 1.50). The overall BPN-PE score was also positive (*M* = 5.21, *SD* = 1.13). Students also reported high levels of perceived enjoyment

**Table 1:** Descriptive statistics for BPN-PE subscales, overall BPN-PE score, and IMI subscales.

Variable	*M*	*SD*	α
Basic psychological needs (BPN-PE) autonomy			
Autonomy	4.86	1.50	0.79
Competence	5.32	1.28	0.80
Relatedness	5.47	1.38	0.83
Overall BPN-PE score	5.21	1.13	0.87
Intrinsic motivation (IMI)			
Perceived enjoyment	5.57	1.49	0.82
Perceived effort	5.54	1.42	0.80

*N* = 188. BPN-PE, basic psychological needs in physical education; IMI, intrinsic motivation inventory; α, Cronbach's alpha.

(*M* = 5.57, *SD* = 1.49) and perceived effort in PE (*M* = 5.54, *SD* = 1.42). According to Nunnally's (1975) threshold of 0.70 for the psychological domain, internal consistency estimates were acceptable across all variable, with Cronbach's alpha values ranging from.79 to.87. Overall, these findings suggest that students perceived their PE experiences positively, particularly in relation to enjoyment, effort, and relatedness.

The analytic samples varied across the three comparison groups. Subgroup selection was based on the availability of the relevant characteristics within the same school among students who completed the surveys. Specifically, for the nationality comparison, only schools in which both Saudi and non-Saudi students completed the survey were included. Similarly, for the grade-level comparison, only schools in which students from both seventh and eighth grades completed the survey were retained. Schools in which only one of the two grade levels was represented among survey respondents, or in which respondents were from only one nationality, were excluded from the respective analyses. Consequently, the nationality analysis included 101 students (65 Saudi and 36 non-Saudi), whereas the grade-level analysis included 79 students (37 seventh-grade and 42 eighth-grade students). In contrast, the weekly PE lesson comparison included the full sample (*N* = 188).

The subsamples also differed from the full sample in their overall BPN-PE scores, with mean scores of 5.58 for the nationality subsample and 4.76 for the grade-level subsample, compared with 5.21 for the full sample. Accordingly, findings from the nationality and grade-level analyses are reported as characteristics of their respective subsamples and should not be generalized to the full sample.

**Q2: Do middle school students' perceptions of basic psychological needs satisfaction, enjoyment, and effort in PE differ according to nationality, grade level, and number of weekly PE lessons?**

To answer the second research question, Welch's independent-samples *t* tests were conducted to examine whether students differed in overall BPN-PE, perceived enjoyment, and perceived effort in PE according to nationality, grade level, and number of weekly PE lessons. Because three outcomes were examined within each comparison set, statistical significance was interpreted using a Bonferroni-adjusted alpha level of 0.017 (0.05/3). Effect sizes were reported using Cohen's *d*. As shown in Table 2, no statistically significant differences were found by nationality or grade level subsamples. For weekly PE lessons, statistically significant differences were found for overall BPNPE and enjoyment.

**Table 2:** Group differences in overall BPN-PE, perceived enjoyment, and perceived effort in PE by nationality, grade level, and weekly PE lessons.

Variable	M (SD)	M (SD)	t	df	p	d	95% CI
Nationality	Saudi (*n* = 65)	Non-Saudi (*n* = 36)					
Overall BPNPE	5.62 (0.88)	5.50 (1.13)	0.58	58.92	0.567	0.13	[−0.32, 0.56]
Enjoyment	6.17 (1.18)	6.03 (1.08)	0.63	77.46	0.533	0.13	[−0.32, 0.60]
Effort	5.81 (1.43)	5.79 (1.29)	0.06	78.66	0.954	0.01	[−0.53, 0.57]
Grade level	7th grade (*n* = 37)	8th grade (*n* = 42)					
Overall BPNPE	4.82 (1.37)	4.71 (1.04)	0.40	66.95	0.692	0.09	[−0.44, 0.66]
Enjoyment	5.14 (1.86)	4.63 (1.35)	1.38	64.93	0.173	0.32	[−0.23, 1.25]
Effort	5.22 (1.64)	5.31 (1.26)	−0.26	67.07	0.795	−0.06	[−0.75, 0.57]
Weekly PE lessons	One lesson (*n* = 79)	Two lessons (*n* = 109)					
Overall BPNPE	4.76 (1.20)	5.54 (0.95)	−4.78	144.22	< 0.001^*^	−0.73	[−1.10, −0.46]
Enjoyment	4.86 (1.62)	6.08 (1.16)	−5.67	133.45	< 0.001^*^	−0.88	[−1.64, −0.80]
Effort	5.27 (1.44)	5.74 (1.38)	−2.27	164.16	0.025	−0.34	[−0.88, −0.06]

Analytic samples differed across comparison sets. BPN-PE, basic psychological need satisfaction in physical education; PE, physical education; *M*, mean; *SD*, standard deviation; *t*, Welch's *t* statistic; *df*, Welch-Satterthwaite degrees of freedom; *p*, two-tailed probability value; *d*, Cohen's *d*. Negative *t* and *d* values indicate higher scores for the group in the right-hand column. *d* values of 0.20, 0.50, and 0.80 were interpreted as small, medium, and large effects, respectively. *p* values were interpreted using a Bonferroni-adjusted alpha level of.017 within each comparison set.

For nationality, Saudi and non-Saudi students reported similar mean scores across all three variables. No statistically significant differences were found for overall BPN-PE, *t*_(58.92)_ = 0.58, *p* = 0.567, *d* = 0.13; perceived enjoyment, *t*_(77.46)_ = 0.63, *p* = 0.533, *d* = 0.13; or perceived effort, *t*_(78.66)_ = 0.06, *p* = 0.954, *d* = 0.01. These findings suggest that Saudi and non-Saudi students reported similar levels of basic psychological needs satisfaction, perceived enjoyment, and perceived effort in PE within the segmented nationality comparison sample.

For grade level, no statistically significant differences were found between 7th- and 8th-grade students. Differences were not significant for overall BPN-PE, *t*_(66.95)_ = 0.40, *p* = 0.692, *d* = 0.09; perceived enjoyment, *t*_(64.93)_ = 1.38, *p* =.173, *d* = 0.32; or perceived effort, *t*_(67.07)_ = −0.26, *p* = 0.795, *d* = −0.06. These findings suggest that 7th- and 8th-grade students reported similar levels of basic psychological needs satisfaction, perceived enjoyment, and perceived effort PE. Given the relatively small subsample (*n* = 79), this finding should be interpreted cautiously as potentially indicating a grade-level difference that the available sample size may have been insufficient to detect with adequate statistical power, rather than as evidence that no difference exists.

For weekly PE lessons, students receiving two PE lessons per week reported higher mean scores than students receiving one PE lesson per week across all three variables. Statistically significant differences were found for overall BPN-PE, *t*_(144.22)_ = −4.78, *p* < 0.001, *d* = −0.73, and enjoyment, *t*_(133.45)_ = −5.67, *p* < 0.001, *d* = −0.88. Although students receiving two PE lessons also reported higher perceived effort, this difference was not statistically significant after Bonferroni correction, *t*_(164.16)_ = −2.27, *p* = 0.025, *d* = −0.34. These findings suggest that students receiving two weekly PE lessons reported higher basic psychological needs satisfaction and enjoyment than students receiving one weekly PE lesson, while the difference in perceived effort in PE did not reach the adjusted significance level.

This pattern should be interpreted with caution. Because weekly PE lesson frequency was determined at the school level rather than assigned to individual students, the comparison may be influenced by unmeasured school-level factors, including facilities, staffing, and program emphasis. In addition, the two groups were substantially unequal in size (*n* = 79 vs. *n* = 109). Thus, the findings indicate an association between greater PE lesson frequency and more positive perceptions of overall BPN-PE and enjoyment; however, they do not demonstrate that increasing the frequency of PE lessons would produce that difference.

**Q3: What are the relationships among basic psychological needs satisfaction, perceived enjoyment, and perceived effort in PE?**

Pearson product-moment correlations were conducted to examine the relationships among overall BPN-PE, perceived enjoyment, and perceived effort in PE. As shown in Table 3, all correlations were positive and statistically significant. Overall BPN-PE was strongly and positively related to enjoyment, *r* = 0.68, *p* < 0.001, and positively related to perceived effort, *r* = 0.57, *p* < 0.001. Enjoyment was also positively related to perceived effort, *r* = 0.62, *p* < 0.001. These findings indicate that students who reported higher basic psychological needs satisfaction in PE also tended to report higher enjoyment and perceived effort.

**Table 3:** Pearson correlations among overall BPN-PE, perceived enjoyment, and perceived effort in PE.

Variable	1	2	3
1. Overall BPN-PE	—		
2. Enjoyment	0.68^***^	—	
3. Perceived effort	0.57^***^	0.62^***^	—

*N* = 188. BPN-PE, basic psychological needs in physical education. Values are Pearson correlation coefficients. ^***^p < 0.001.

## 4 Discussion

This study examined middle school students' perceptions of autonomy, competence, relatedness, overall BPN-PE, perceived enjoyment, and perceived effort in Saudi Arabian PE, as well as whether these perceptions varied by nationality, grade level, and weekly PE lesson frequency. Students generally reported high levels of relatedness, competence, enjoyment, and effort, while autonomy was rated lower than the other basic psychological needs. These findings are important because they show how effectively the Saudi PE environment supports students' motivational experiences, as described by Self-Determination Theory (Ryan and Deci, 2017). The findings highlight the strengths of the Saudi PE context in supporting relatedness, competence, enjoyment, and effort, while identifying autonomy as an area for growth.

The high levels of relatedness, competence, and enjoyment observed in this study may be explained by several complementary factors. First, PE is consistently regarded as one of the most enjoyable school subjects by many students. Previous research has shown that ~80% of students report positive attitudes toward PE and enjoy participating in lessons (Carlson, 1995; Ryan et al., 2003). Such positive affective responses are likely to contribute to higher perceptions of enjoyment and engagement. Second, PE offers opportunities that extend beyond physical activity itself. For many students, lessons provide a valuable social environment in which they can interact with peers, collaborate in activities, and experience a temporary departure from the academic demands of the classroom. Consequently, students' positive perceptions may reflect not only the instructional content of PE but also the broader social and recreational experiences embedded within the lesson context. Finally, the traditional PE curriculum commonly implemented in Saudi schools provides students with repeated opportunities to practice motor skills during each lesson, which may strengthen perceptions of competence through continual skill development and successful task performance (Alshuraymi and Hastie, 2025).

Despite these positive findings, autonomy emerged as the least satisfied of the three basic psychological needs. This result is likely associated with the predominant use of traditional instructional practices in Saudi Arabian PE. Traditional approaches are typically teacher-centered and content-driven, with teachers determining lesson objectives, instructional activities, organizational structures, and student roles. Within such learning environments, students generally have limited opportunities to make meaningful choices, participate in decision-making, or influence the learning process (Metzler, 2017). According to Self-Determination Theory, the absence of autonomy-supportive teaching practices can undermine students' intrinsic motivation and reduce the quality of their learning experiences (Ryan and Deci, 2017). Similarly, instructional models characterized by high levels of teacher control have been shown to restrict student engagement and diminish motivational outcomes in PE (Metzler, 2017). Therefore, although the current PE environment appears effective in supporting students' feelings of competence and relatedness, it may be less successful in fostering students' sense of autonomy.

A recent study by Alshuraymi and Hastie (2025) examined the implementation of the Sport Education instructional model in Saudi Arabian middle school PE by comparing its effects with those of Traditional PE. Specifically, the study investigated the extent to which each instructional approach influenced students' intrinsic motivation and their perceived achievement of the Saudi national objectives for PE. Findings from that study indicated that students who participated in Sport Education demonstrated significant improvements in all three basic psychological needs, as well as perceived enjoyment and perceived effort, following the intervention. In contrast, students receiving Traditional PE showed improvements only in competence and enjoyment. Collectively, these findings suggest that although the current Saudi PE context provides a generally positive learning environment, instructional approaches that intentionally promote student autonomy, shared decision-making, authentic roles, and sustained team affiliation, such as Sport Education, may offer substantially greater benefits for satisfying students' basic psychological needs and strengthening motivational outcomes. These findings further support the growing body of evidence indicating that learner-centered pedagogical models are more effective than traditional teacherdirected instruction in promoting intrinsically motivating learning environments in PE (Alshuraymi and Hastie, 2025).

These findings should also be considered within the broader context of ongoing educational reform in Saudi Arabia. The launch of Saudi Vision 2030 Saudi Vision (2030, 2016) has placed increasing emphasis on improving citizens' health, wellbeing, and quality of life through comprehensive reforms across multiple sectors, including education (KSA Government, 2016; Mohiuddin et al., 2023). As part of this national transformation, the Education and Training Evaluation Commission (2020) introduced a set of professional standards for health and PE teachers, which have guided the development of a contemporary PE curriculum aimed at promoting students' physical, psychological, and social development through holistic and competency-based learning experiences. Despite these curriculum reforms and policy-level advancements, instructional practices within Saudi PE settings appear to have evolved more gradually. Many PE teachers continue to rely predominantly on traditional, teacher-centered approaches that provide limited opportunities for students to exercise choice, participate in decision-making, and take ownership of their learning. Therefore, the lower autonomy scores observed in the present study may indicate a gap between the objectives of recent PE curriculum reforms and the instructional practices currently implemented in PE settings, highlighting the need for greater opportunities for student choice, responsibility, and decision-making.

This gap highlights an important implication for PE practice and teacher development in Saudi Arabia. To fully realize the objectives of Vision 2030 and the national PE curriculum, professional development initiatives should support teachers in transitioning toward more autonomy-supportive and student-centered pedagogies. Instructional models such as Sport Education (Siedentop, 1994) may provide a practical framework for achieving this goal by promoting meaningful student roles, shared responsibility, decision-making opportunities, and stronger social connections within PE lessons. The emerging evidence from Alshuraymi and Hastie (2025) and Alshuraymi et al. (2026) suggests that such approaches can enhance students' basic psychological need satisfaction, intrinsic motivation, engagement, and meaningful learning experiences. Therefore, expanding the implementation of evidence-based learner-centered models, alongside appropriate teacher training and ongoing support, represents an important step toward developing a PE environment that better reflects the educational aspirations of Saudi Vision 2030 and supports students' long-term physical activity motivation and wellbeing.

## 5 Limitations

Several limitations should be considered when interpreting the findings of the present study. First, the cross-sectional nature of the study limits the ability to draw causal conclusions regarding students' basic psychological needs, perceived enjoyment, and perceived effort. Although the findings provide an important snapshot of students' motivational experiences in Saudi Arabian PE, longitudinal and experimental designs are needed to examine how these perceptions develop over time and how they may respond to changes in instructional practices.

Second, the study was conducted exclusively in middle schools in Riyadh, Saudi Arabia, and included only 188 students from all-boys schools. The relatively small and geographically specific sample limits the generalizability of the findings to middle school students across other regions of Saudi Arabia. In addition, because the sample consisted exclusively of male students, the findings cannot be assumed to represent female students' experiences in PE. Future research should therefore include larger and more diverse samples drawn from multiple regions of Saudi Arabia and from both boys' and girls' schools to provide a more comprehensive understanding of students' motivational experiences across different educational contexts.

Third, the study relied primarily on students' self-reported perceptions, which may be influenced by social desirability, individual response tendencies, or differences in how students interpret questionnaire items. Future studies could strengthen the assessment of students' motivational experiences by incorporating multiple sources of evidence, including systematic observations of PE lessons, teacher reports, student interviews, or other qualitative approaches.

Fourth, although the study examined differences according to nationality, grade level, and weekly PE lesson frequency, other contextual factors that may influence students' motivational experiences were not examined. These factors may include teacher characteristics and experience, class size, school-level characteristics, available facilities and resources, and the specific instructional approaches or pedagogical models used by PE teachers. Future research should consider these variables to develop a more comprehensive understanding of the factors associated with basic psychological need satisfaction, perceived enjoyment, and perceived effort in Saudi PE.

Finally, the findings should be interpreted within the specific educational and cultural context of Riyadh middle schools. Although the study contributes valuable evidence regarding students' motivational experiences in Saudi Arabian PE, differences in school environments, regional contexts, instructional practices, and student populations may produce different patterns of results elsewhere. Replication across multiple Saudi regions, educational levels, and student populations is therefore warranted to determine the broader applicability of the present findings.

## 6 Conclusion

The present study provides important insight into middle school students' motivational experiences in physical education in Saudi Arabia through the framework of Self-Determination Theory. Overall, students reported relatively high levels of relatedness, competence, perceived enjoyment, and perceived effort, whereas autonomy was comparatively lower. These findings suggest that, while students generally experience positive motivational and affective outcomes in PE, greater attention may be needed to provide opportunities for student choice, decision-making, and ownership of the learning process.

The findings have important implications for the continued development of PE in Saudi Arabia. As the national education system continues to emphasize students' holistic development, health, and wellbeing through Saudi Vision 2030, PE teachers may benefit from greater support in adopting autonomy-supportive and student-centered instructional practices. Approaches such as Sport Education, which provide students with meaningful roles, responsibility, decision-making opportunities, and sustained social interaction, may offer a promising avenue for addressing students' psychological needs and enhancing their motivational experiences.

Although the present findings provide a useful snapshot of students' perceptions, further research using larger and more diverse samples, longitudinal designs, and intervention-based approaches is needed to examine how instructional practices influence students' basic psychological needs, enjoyment, and effort over time. Continued attention to autonomy-supportive teaching may help create more meaningful and motivating PE environments and contribute to the broader educational and health-related goals of PE in Saudi Arabia.

## Data Availability

The raw data supporting the conclusions of this article will be made available by the authors, without undue reservation.
